# 551 Reduction of Fungus-Related Complications in a Burn Center

**DOI:** 10.1093/jbcr/irad045.148

**Published:** 2023-05-15

**Authors:** Sarah Shingleton, Kristine Chafin, Garrett Britton, James Aden, Anthony Basel, Leopoldo Cancio

**Affiliations:** US Army Institute of Surgical Research, San Antonio, Texas; US Army Institute of Surgical Research, San Antonio, Texas; US Army Institute of Surgical Research, San Antonio, Texas; Brooke Army Medical Center, San Antonio, Texas; Brooke Army Medical Center, San Antonio, Texas; US Army Institute of Surgical Research, San Antonio, Texas; US Army Institute of Surgical Research, San Antonio, Texas; US Army Institute of Surgical Research, San Antonio, Texas; US Army Institute of Surgical Research, San Antonio, Texas; Brooke Army Medical Center, San Antonio, Texas; Brooke Army Medical Center, San Antonio, Texas; US Army Institute of Surgical Research, San Antonio, Texas; US Army Institute of Surgical Research, San Antonio, Texas; US Army Institute of Surgical Research, San Antonio, Texas; US Army Institute of Surgical Research, San Antonio, Texas; Brooke Army Medical Center, San Antonio, Texas; Brooke Army Medical Center, San Antonio, Texas; US Army Institute of Surgical Research, San Antonio, Texas; US Army Institute of Surgical Research, San Antonio, Texas; US Army Institute of Surgical Research, San Antonio, Texas; US Army Institute of Surgical Research, San Antonio, Texas; Brooke Army Medical Center, San Antonio, Texas; Brooke Army Medical Center, San Antonio, Texas; US Army Institute of Surgical Research, San Antonio, Texas; US Army Institute of Surgical Research, San Antonio, Texas; US Army Institute of Surgical Research, San Antonio, Texas; US Army Institute of Surgical Research, San Antonio, Texas; Brooke Army Medical Center, San Antonio, Texas; Brooke Army Medical Center, San Antonio, Texas; US Army Institute of Surgical Research, San Antonio, Texas; US Army Institute of Surgical Research, San Antonio, Texas; US Army Institute of Surgical Research, San Antonio, Texas; US Army Institute of Surgical Research, San Antonio, Texas; Brooke Army Medical Center, San Antonio, Texas; Brooke Army Medical Center, San Antonio, Texas; US Army Institute of Surgical Research, San Antonio, Texas

## Abstract

**Introduction:**

Fungal wound infections (FWI) cause morbidity and increase mortality in burn patients. Our burn center experienced 44 patients with fungal wound colonization (FWC) and/or FWI between JAN 2015 and JAN 2019. In response, we undertook a performance improvement project to prevent and treat FWC and FWI.

**Methods:**

Members of the multidisciplinary team met to develop a clinical practice guideline (CPG) for the prevention and management of FWC and FWI based on current evidence. We focused on patients with elevated risk, that is, with burns ≥20% total body surface area (TBSA) and in the Burn ICU (BICU). Interventions included: utilizing alternating silver sulfadiazine and mafenide acetate creams upon admission; reducing the use of mafenide acetate solution; utilizing silver nitrate solution post-operatively; and applying topical nystatin cream or powder for suspected FWC or FWI. We educated all staff members and updated order sets and training materials. We collected data on all burn patients who had a wound biopsy out of concern for possible infection. Biopsy results were categorized as FWC or FWI. Retrospective data were collected for MAR 2020 - MAR 2021 (PRE). Post-implementation, prospective data collection began MAR 2022 (POST) and will continue for one year. Adherence to the CPG was assessed by chart review. Mann-Whitney and Fischer Exact tests were performed.

**Results:**

The PRE (n=15) and POST (n=9) groups were similar in age (43±13 vs. 48±18 years) but differed in TBSA (49±19 vs 28±25%, p< .05). PRE group biopsies showed FWC in 0 patients and FWI in 11 of 15 patients (73%); 8 of these (53%), all with FWI, died. In the POST group, FWC was found in 1 patient (11%) and FWI was found in 2 of 9 patients (22%); one death occurred in the patient with FWC. Adherence to the CPG for admission topical wound care was 7% in the PRE group vs 89% in the POST group (p=0.0001); adherence to the CPG for day-of-biopsy topical wound care was 27% in the PRE group vs 89% in the POST group (p< 0.01).

**Conclusions:**

Adherence to a CPG for the prevention and treatment of FWC and FWI was associated with a lower (but not statistically significant) prevalence of these complications. Limitations include a difference in the TBSA between the 2 groups and potential concurrent changes in practice. Ongoing data collection includes the evaluation of other potential contributing factors.

**Applicability of Research to Practice:**

Fungi are common in the environment, and when combined with immunosuppression and extensive open wounds, may cause wound infection in burn patients. More research is needed to further evaluate effective treatments for the prevention and treatment of FWC and FWI in these patients.

